# Molecular Mechanism of 5,6-Dihydroxyflavone in Suppressing LPS-Induced Inflammation and Oxidative Stress

**DOI:** 10.3390/ijms251910694

**Published:** 2024-10-04

**Authors:** Yujia Cao, Yee-Joo Tan, Dejian Huang

**Affiliations:** 1 Department of Food Science and Technology, National University of Singapore, Singapore 117542, Singapore; yujia.cao@u.nus.edu; 2 Infectious Diseases Translational Research Program, Department of Microbiology and Immunology, Yong Loo Lin School of Medicine, National University of Singapore, Singapore 117545, Singapore; mictyj@nus.edu.sg; 3 National University of Singapore (Suzhou) Research Institute, 377 Linquan Street, Suzhou 215123, China

**Keywords:** 5,6-dihydroxyflavone, flavonoids, anti-inflammation, oxidative stress, signaling pathway

## Abstract

5,6-dihydroxyflavone (5,6-DHF), a flavonoid that possesses potential anti-inflammatory and antioxidant activities owing to its special catechol motif on the A ring. However, its function and mechanism of action against inflammation and cellular oxidative stress have not been elucidated. In the current study, 5,6-DHF was observed inhibiting lipopolysaccharide (LPS)-induced nitric oxide (NO) and cytoplasmic reactive oxygen species (ROS) production with the IC_50_ of 11.55 ± 0.64 μM and 0.8310 ± 0.633 μM in murine macrophages, respectively. Meanwhile, 5,6-DHF suppressed the overexpression of pro-inflammatory mediators such as proteins and cytokines and eradicated the accumulation of mitochondrial ROS (mtROS). The blockage of the activation of cell surface toll-like receptor 4 (TLR4), impediment of the phosphorylation of c-Jun N-terminal kinase (JNK) and p38 from the mitogen-activated protein kinases (MAPK) pathway, Janus kinase 2 (JAK2) and signal transducer and activator of transcription 3 (STAT3) from the JAK-STAT pathway, and p65 from nuclear factor-κB (NF-κB) pathways were involved in the process of 5,6-DHF suppressing inflammation. Furthermore, 5,6-DHF acted as a cellular ROS scavenger and heme-oxygenase 1 (HO-1) inducer in relieving cellular oxidative stress. Importantly, 5,6-DHF exerted more potent anti-inflammatory activity than its close structural relatives, such as baicalein and chrysin. Overall, our findings pave the road for further research on 5,6-DHF in animal models.

## 1. Introduction

Inflammation is a complicated immune response to defense noxious foreign agents and internal abnormal conditions [1]. Inflammation-derived oxidative stress that basically attributes to the reactive oxygen species (ROS) leads to dangerous damages on cellular essential components such as lipids, proteins, and DNA and further aggravates the pathological inflammation [2,3]. Also, the positive feedback loop between inflammation and oxidative stress has been established [4]. 

Flavonoids are an important family of polyphenolic compounds belonging to plant secondary metabolites and widely distributed in fruits, vegetables, and medicinal plants [5]. Flavonoids have been reported to have a variety of health-promoting effects, including antioxidant, anti-inflammatory, and anti-tumor properties [6]. In particular, flavonoids have strong influence on the inflammatory processes with diverse identified mechanisms, such as the nuclear factor kappa-B (NF-κB) pathway, the mitogen-activated protein kinase (MAPK) pathway, and the Janus kinase (JAK)-signal transducer and activator of the transcription (STAT) pathway, that are attributable to their structural and functional diversity [6,7,8]. For example, catecholic moieties played an indispensable role as a functional group for the anti-inflammatory activity of flavonoids [9]. As a class of polyphenols, flavonoids widely receive attention for their antioxidant functions as radical scavengers [10]. Apart from numerous flavonoids isolated from natural resources, chemically synthesized or structurally modified flavonoids have also promoted the development of research on the biological and pharmacological activity of flavonoids to a certain extent [11].

**Figure 1:**
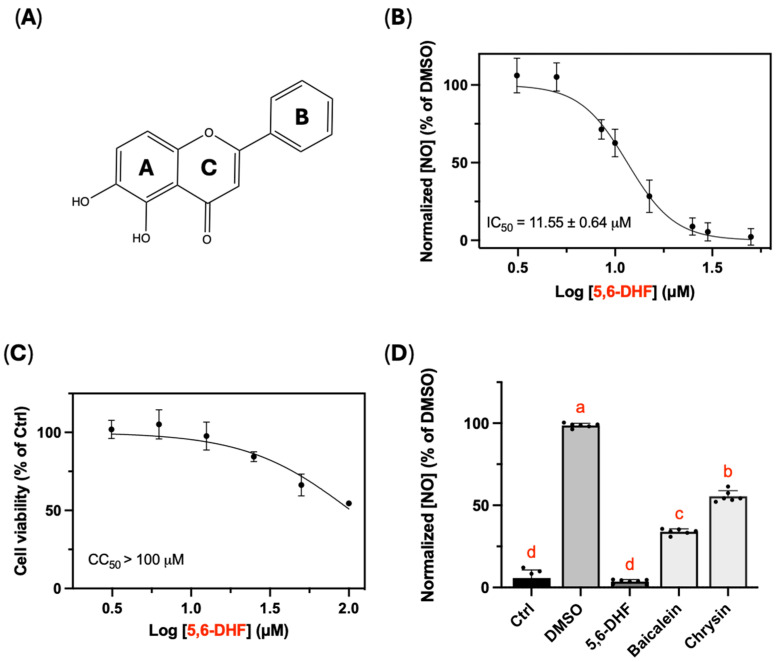
Chemical structure of 5,6-DHF (**A**). Dose response curves of 5,6-DHF on LPS-induced NO production (**B**) and cell cytotoxicity of 5,6-DHF on RAW 264.7 cells (**C**). Comparison of the anti-NO activity of 5,6-DHF and its structural analogues at 25 μM (**D**). The values shown are the mean ± SD of three independent experiments in duplicate. Different letters indicate statistical significance (*p* < 0.05).

5,6-dihydroxyflavone (5,6-DHF), a rare flavonoid aglycone found in nature, is obtained commercially through chemical synthesis as there are few reports on its natural occurrence [12]. It has been found in rat urine after oral administration of the traditional Chinese medicine formula Xiao-Ai-Jie-Du, decoction which is prescribed to reduce fever, detoxify, and resolve phlegm and congestion [13]. Possessing the catechol motif at the A ring (Figure 1A), 5,6-DHF showed radical scavenging activity with a Trolox equivalent antioxidant capacity (TEAC) of 0.98 ± 0.05 μM [14]. Results from intracellular fluorescent probes and electron paramagnetic resonance spectroscopy revealed the intercellular antioxidant effect of 5,6-DHF by eliminating ROS in L-6 and THP-1 cells at concentrations as low as 100 pM [15]. Additionally, its potential utilization in treating Alzheimer’s disease, a neuroinflammatory disease, was demonstrated with a half-maximal inhibitory concentration (IC_50_) of 4.51 ± 0.03 μM on acetylcholinesterase (AChE), which is a cholinergic enzyme in the nervous system [14]. Collectively, 5,6-DHF holds high potency in modulating cellular inflammatory responses and oxidative stress due to its anti-inflammatory and antioxidant function, yet its performance on immune cells in inflammatory conditions and its underlying mechanisms of action have nevertheless not been elucidated. Herein, we investigated the effects of 5,6-DHF on lipopolysaccharide (LPS)-induced inflammation and oxidative stress in RAW 264.7 cells and elucidated its mechanisms of action.

## 2. Results

### 2.1. Effect of 5,6-DHF on LPS-Activated Nitric Oxide Production

To preliminarily screen the inhibitory effects of 5,6-DHF on LPS-activated inflammation in RAW 264.7 cells, the nitric oxide (NO) concentration in the cell culture medium was measured. Figure 1B showed that 5,6-DHF significantly attenuated the secreted NO from LPS-induced RAW 264.7 cells in a dose-dependent manner. The preliminary results demonstrated 5,6-DHF was a potential anti-inflammatory candidate that inhibited LPS-induced NO production with an IC_50_ of 11.55 ± 0.64 μM (Figure 1B). In addition, 5,6-DHF slightly impacted cell growth in a dose-dependent manner, but the cytotoxic concentration (CC_50_) was greater than 100 μM (Figure 1C). Interestingly, compared with its structural analogues, baicalein and chrysin, 5,6-DHF exerted significantly high potency on the suppression of NO at the concentration of 25 μM, which is about twice the IC_50_ of 5,6-DHF.

### 2.2. Suppressive Effects of 5,6-DHF on Key Inflammatory-Related Proteins and Pro-Inflammatory Cytokines

**Figure 2:**
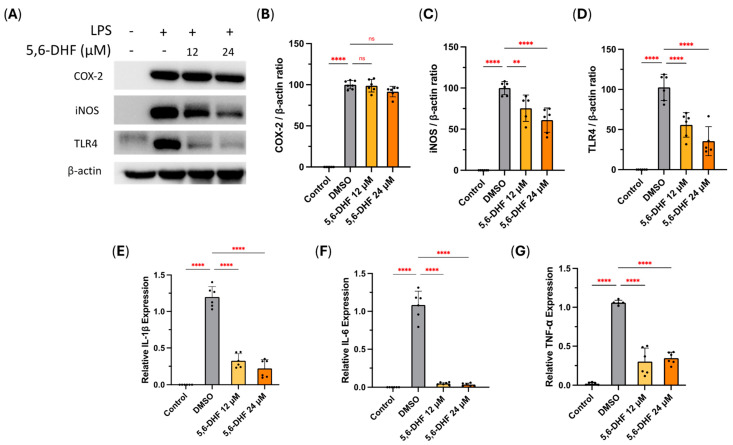
The suppressive effects of 5,6-DHF on pro-inflammatory protein expression in LPS-stimulated RAW 264.7 models (**A**). The expression levels of COX-2 (**B**), iNOS (**C**), and TLR4 (**D**) were determined by Western blot. The inhibitory effects of 5,6-DHF on mRNA expression levels of IL-1β (**E**), IL-6 (**F**), and TNF-α (**G**) tested by qRT-PCR. Data points and bar represent arithmetic means ± SD. Ns, not significant. ** *p* < 0.01 and **** *p* < 0.0001 as compared to DMSO group.

To further confirm the anti-inflammatory effect of 5,6-DHF, we assessed its impact on inflammatory-related proteins including cyclooxygenase-2 (COX-2), inducible nitric oxide synthase (iNOS), and toll-like receptor 4 (TLR4) at its IC_50_ against NO production and twice the amount of IC_50_ using Western blotting (Figure 2A). We found 5,6-DHF could not alleviate the LPS-induced expression of COX-2 in RAW 264.7 cells (Figure 2B). In contrast, 5,6-DHF significantly hindered the expression of iNOS (Figure 2C). We also evaluated the role that 5,6-DHF played in the interaction between LPS and cell surface recognitive receptors. Figure 2D indicates that 5,6-DHF could significantly block the LPS-induced activation of TLR4 expression on the cell surface, therefore protecting RAW 264.7 cells from stimulation by foreign agents.

Furthermore, the alterations of three predominant pro-inflammatory cytokines, interleukin (IL)-1β, IL-6, and tumor necrosis factor-alpha (TNF-α), were analyzed at 5,6-DHF’s IC_50_ against NO production and twice the amount of IC_50_. The mRNA expression of these pro-inflammatory cytokines in RAW 264.7 macrophages dramatically increased after LPS stimulation. However, the treatment of 5,6-DHF with cells at both 12 and 24 μM could significantly resolve this phenomenon (Figure 2E–G).

### 2.3. Effects of 5,6-DHF on MAPK Pathway

**Figure 3:**
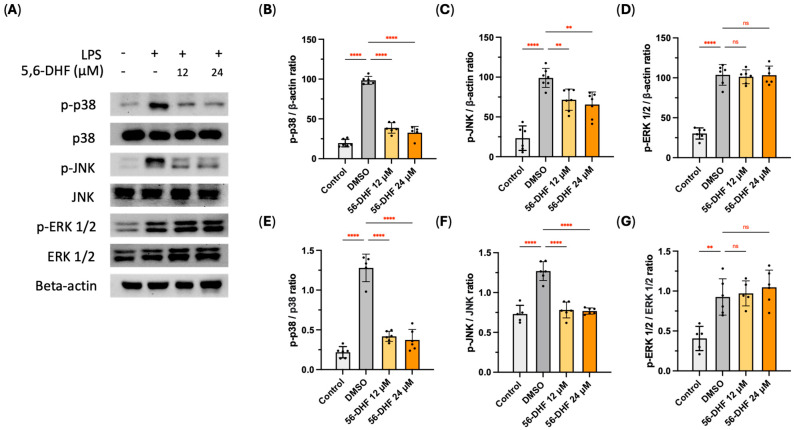
Effects of 5,6-DHF on MAPK pathway in LPS-induced RAW 264.7 cells (**A**). Suppressive effects of 5,6-DHF on the LPS-induced p-p38 (**B**), p-JNK, (**C**) and p-ERK1/2 (**D**) expressions and the phosphorylation level of p38 (**E**), JNK (**F**), and ERK1/2 (**G**). All expressions were normalized to that of DMSO treatment. Data points and bar represent arithmetic means ± SD. Ns, not significant. ** *p* < 0.01 and **** *p* < 0.0001 as compared to DMSO group.

The activation of MAPKs is recognized by the phosphorylation of three families of proteins, namely extracellular signal-regulated kinase 1/2 (ERK1/2), p38 MAPK, and c-Jun N-terminal kinase (JNK) [16]. As Figure 3A–D illustrates, LPS triggered a significant phosphorylation of ERK1/2, p38, and JNK as compared with the non-treated control group, yet 5,6-DHF downregulated the expression level of phosphorylated typed p38 and JNK. By calculating the ratio of phosphorylated MAPKs to their prototype ones, we noticed 5,6-DHF obstructed the phosphorylation process of p38 and JNK, thereby blocking the detrimental actions of MAPKs in the inflammatory response cascade (Figure 3E–G).

### 2.4. Effects of 5,6-DHF on JAK-STAT Pathway

**Figure 4:**
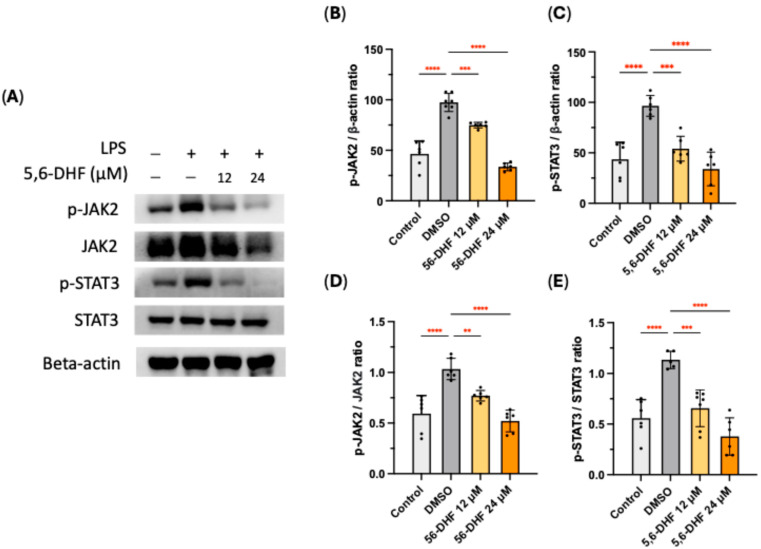
Effects of 5,6-DHF on JAK-STAT pathway in LPS-induced RAW 264.7 cells (**A**). Suppressive effects of 5,6-DHF on the LPS-induced p-JAK2 (**B**) and p-STAT3 (**C**) expression and the phosphorylation level of JAK2 (**D**) and STAT3 (**E**). All expressions were normalized to that of DMSO treatment. Data points and bar represent arithmetic means ± SD. Ns, not significant. ** *p* < 0.01, *** *p* < 0.001, and **** *p* < 0.0001 as compared to DMSO group.

The influence of 5,6-DHF on the JAK-STAT pathway was analyzed by measuring the phosphorylation expression of key proteins (Figure 4A). Cells stimulated by LPS showed an obvious increment in the expression of phosphorylated JAK2 and STAT3 levels. 5,6-DHF reversed this trend in a concentration-dependent manner (Figure 4B,C). In comparison with the untreated group, 5,6-DHF significantly reduced the LPS-induced phosphorylation ratios of JAK2 and STAT3 (Figure 4D,E).

### 2.5. Effects of 5,6-DHF on NF-κB Pathway

**Figure 5:**
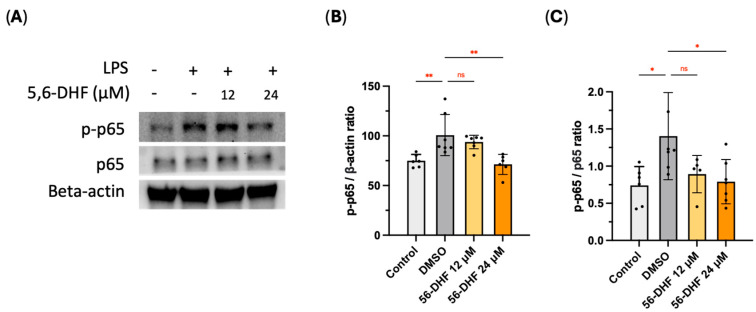
Effects of 5,6-DHF on NF-κB pathway in LPS-induced RAW 264.7 cells (**A**); suppressive effects of 5,6-DHF on the LPS-induced p-p65 expression (**B**); and the phosphorylation level of p65 (**C**). All expressions were normalized to that of DMSO treatment. Data points and bar represent arithmetic means ± SD. Ns, not significant. * *p* < 0.05 and ** *p* < 0.01 as compared to DMSO group.

The phosphorylation of the p65 (p-p65) subunit belonging to the NF-κB family in particular marks NF-κB activation by translocating to the nucleus and triggering target gene expressions. To investigate the influence of 5,6-DHF on the NF-κB pathway, the expression and phosphorylation level of p65 were determined (Figure 5). There was no difference in activation levels between cells incubated with a low dose (12 μM) of 5,6-DHF and those incubated with DMSO, whereas a higher dose (24 μM) of 5,6-DHF successfully hindered the activation of NF-κB, showing significantly suppressive effects on the p-p65 expression and phosphorylation level of p65 (Figure 5B,C).

### 2.6. Effect of 5,6-DHF on LPS-Induced Cytoplasmic and Mitochondrial ROS

**Figure 6:**
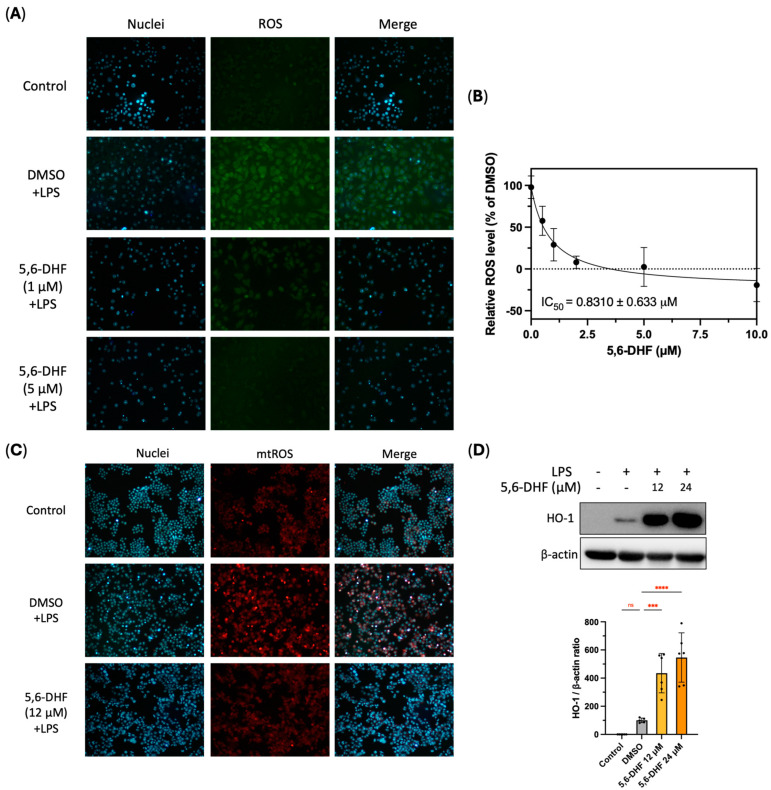
Cellular ROS-scavenging activity of 5,6-DHF on LPS-induced RAW264.7 cells, images were captured by fluorescent microscope (**A**); blue: cell nuclei stained by H33342; and green: cellular ROS stained by H_2_DCFDA. Dose-response curve of ROS-scavenging activity of 5,6-DHF; scale bar 20 μm (**B**). Mitochondrial ROS-scavenging activity of 5,6-DHF on LPS-induced RAW264.7 cells (**C**). The fluorescence was visualized by fluorescent microscopy (blue: cell nuclei stained by H33342; and red: mitochondrial ROS stained by MitoSOX red). The influence of 5,6-DHF on the expression levels of HO-1; scale bar 20 μm (**D**) Data points and bar represent arithmetic means ± SD. Ns, not significant. *** *p* < 0.001 and **** *p* < 0.0001 as compared to DMSO group.

Since 5,6-DHF is a radical scavenger, its capability to inhibit LPS-induced oxidative stress in RAW 264.7 cells was first investigated by measuring intracellular ROS levels that predominantly originated from NADPH oxidases (Figure 6A). Our previous study showed 1 μM flavones could reduce intracellular ROS levels caused by tert-butyl hydroperoxide (tBHP) [17]. We observed that LPS led to intensive accumulation of ROS signal in the intercellular region of cells treated with DMSO, while cells incubated with 1 μM 5,6-DHF remarkedly attenuated cytoplasmic ROS signal (Figure 6A). Meanwhile, the 5 μM 5,6-DHF group exhibited a negligible ROS signal compared to that of the positive control group, demonstrating its ROS scavenging activity. Dose-dependent manner of 5,6-DHF on scavenging LPS-induced ROS was revealed, and its IC_50_ value was 0.8310 ± 0.633 μM (Figure 6B).

In addition to cytoplasmic ROS, the alteration of mitochondrial ROS (mtROS) characterized by superoxide in live cells was also visualized by a redox-sensitive fluorescent probe, MitoSOX red. LPS produced a strong oxidative stress in the DMSO group in RAW 264.7 cells, while the mtROS content in the 12 μM 5,6-DHF group was significantly lower, indicating that 5,6-DHF attenuated the overproduction of mtROS (Figure 6C).

### 2.7. 5,6-DHF Exhibited Antioxidant Activity by Inducing HO-1 Expression

To understand the other mechanism of 5,6-DHF suppressing cellular oxidative stress, the impact of 5,6-DHF on heme oxygenase 1 (HO-1) expression was assessed in RAW 264.7 cells. Remarkably, 5,6-DHF significantly induced the generation of HO-1 at both tested concentrations, suggesting its function in counteracting LPS-caused cellular oxidative stress in macrophages (Figure 6D).

**Figure 7:**
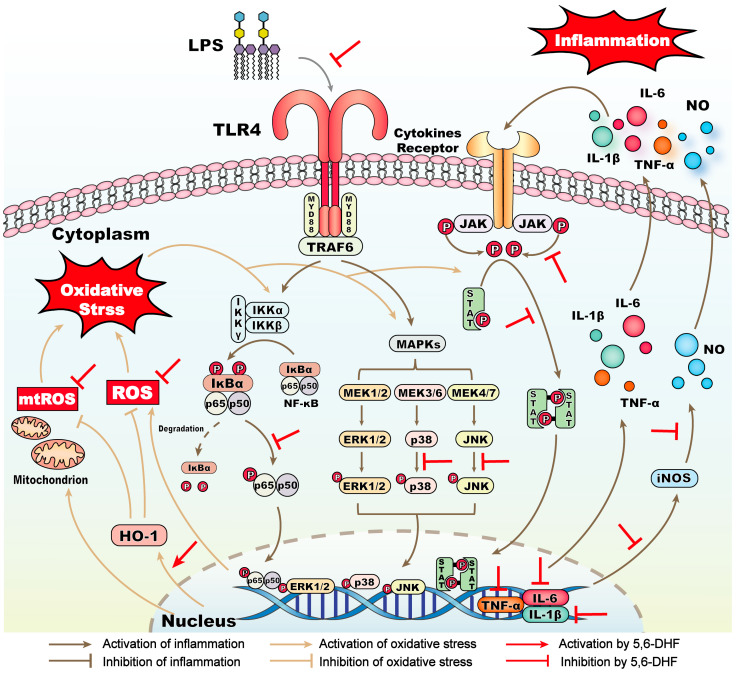
Schematic diagram of 5,6-DHF mechanisms in inhibiting LPS-induced inflammatory responses and oxidative stress in RAW 264.7 cells.

Taken together, the overall mechanisms of 5,6-DHF on relieving LPS-induced inflammatory response and oxidative stress are summarized in Figure 7. Specifically, 5,6-DHF inhibited LPS-induced activation of TLR4 and phosphorylation of p38 and JNK in the MAPK pathway, JAK2 and STAT3 in the JAK-STAT pathway, and p65 in the NF-κB pathways. Its role in suppressing cytokine expression, including IL-1β, IL-6, and TNF-α, and downregulating downstream pro-inflammatory mediators such as iNOS and NO in murine macrophages is also attributed to its anti-inflammatory activity. The reduction of LPS-induced cellular oxidative stress by 5,6-DHF was manifested by scavenging cytoplasmic ROS and mtROS. The induction of HO-1 by 5,6-DHF was also responsible for protecting cells from oxidative damage.

## 3. Discussion

Inflammation disturbs the intercellular redox equilibrium, resulting in uncontrollable oxidation of pivotal cellular components and severe inflammation [4]. Flavonoids, as a class of polyphenolic compounds, have earned a favorable reputation regarding their anti-inflammatory and antioxidant properties [18]. Particularly, the catechol moiety at the A ring renders 5,6-DHF a promising contribution to resolving inflammation and eradicating cellular oxidative radicals [19].

The inflammatory mediator NO is a clinical biomarker of several inflammatory diseases [20]. It is biosynthesized under the catalysis of iNOS under harmful stimulation. 5,6-DHF was discovered to attenuate the expression of iNOS, thus prohibiting the production of NO. However, unlike commonly prescribed conventional nonsteroidal anti-inflammatory drugs (NSAIDs) that target COX-2, 5,6-DHF could not diminish COX-2 expression level in RAW 264.7 cells. In addition, dysregulated pro-inflammatory cytokine production contributes to the pathogenesis of inflammation-related disorders like neurodegenerative diseases, cardiovascular disease, and cancer [21,22,23]. We observed macrophage-derived pro-inflammatory cytokines, such as IL-1β, IL-6, and TNF-α, tumbled down with the co-treatment of 5,6-DHF under LPS-activated circumstances, therefore relieving inflammation.

Surface receptor, TLR4, specifically recognizes LPS upon stimulation and triggers the activation of molecular determinants in the LPS-induced signaling cascade, including MAPK, JAK-STAT, and NF-κB pathways, resulting in the translocation of mediating factors to the nucleus, where they activate gene transcription and signal transduction [24,25,26,27]. Importantly, MAPK, JAK-STAT, and NF-κB pathways are different, but they interconnect in regulating cellular responses to stimulants such as LPS. Preventing the interaction of LPS and TLR4, 5,6-DHF diminished the activation of surface TLR4 and restrained the following cellular responses to LPS stimulation. The inflammatory signal transduction is marked principally by protein phosphorylation as post-translational modification (PTM) [28]. The phosphorylation levels of predominant markers in MAPK pathway, JAK-STAT pathway, and NF-κB pathway were downregulated by 5,6-DHF treatment, therefore stopping unwanted inflammatory responses. 

It has been established that chronic oxidative stress is a causative factor for aging and age-related diseases such as type II diabetes and chronic kidney disease [29,30,31]. The production of ROS, including superoxide, hydrogen peroxide, and hydroxyl radicals commonly happens in the cytoplasm, mitochondria, and endoplasmic reticulum [28]. Mitochondrion, the energy-producing organelle, contributes the most to the cellular ROS level through the electron transport chain [32]. And ROS derived by electron transfer in the NADPH-oxidase (NOX) bound to cytoplasm membranes is the other prime source for oxidative stress [33]. By clearing both cytoplasmic ROS and mtROS, 5,6-DHF could eliminate oxidative stress induced by LPS and impede unfavorable cell damage. 

The attenuated cytoplasmic ROS and mtROS levels could be credited to the radical scavenging property of 5,6-DHF and its regulatory roles on cellular expression of proteins related to inflammation. Under the condition of LPS stimulation, HO-1, an enzyme that is involved in diverse protective actions for redox balance, was considerably expressed in response to 5,6-DHF treatment in the fight against oxidative stress. Given that excessive ROS is an inducer of inflammation, the cellular oxidative stress orchestrates MAPK, JAK-STAT, and NF-κB pathways and then leads to the production of pro-inflammatory cytokines [34,35,36]. Thus, the impact of 5,6-DHF on these signaling pathways could also hinder the activation of inflammation caused by oxidative stress. 

It is worth pointing out that antioxidant and anti-inflammatory studies of flavonoids primarily lie in flavonoids found in nature because they can be safely consumed as coexisting with the food containing them. Nevertheless, they are only generally safe in low concentrations in natural foods since isolated and concentrated flavonoid compounds have shown cytotoxicity and elevated cellular ROS levels in normal human cells, which hinders their long-term application and weakens their safety advantages [37]. Flavonoids are privileged structural motif for medicinal molecules. In particular, chemical modifications of the structures of flavonoids sheds light on their structure-activity relationship (SAR) [9]. Compared to its natural structural analogues, baicalein and chrysin, 5,6-DHF with modification on the hydroxyl groups in the A ring had the highest potency in blocking inflammatory responses in RAW 264.7 cells. This pinpointed the importance of the catechol motif (5,6-dihydroxyl group) in the A-ring of flavones for their bioactivity. The finding of the predominant role of catechol motif was consistent with our previous study [8]. It is worth mentioning that 5,6-DHF had a CC_50_ greater than 100 μM on RAW 264.7 cells and did not show significant cytotoxicity on myoblasts and monocytes at the concentration that 5,6-DHF exerted its antioxidant effect [15].

To take advantage of the biological and pharmaceutical functions of flavonoids, extensive explorations of structurally different flavonoids will contribute to the optimization of drug or dietary supplement candidates. Also, the combinational use of flavonoids or combining flavonoids with conventional therapeutic agents should be taken into consideration to improve therapeutic efficacy and drug resistance [38,39,40].

## 4. Materials and Methods

### 4.1. Materials

5,6-dihydroxyflavone (purity: 98%) was purchased from Indofine Chemical Co., Inc., Hilsborough, NJ, USA. Chrysin (5,7-dihydroxyflavone, purity: 98%) was supplied by Shanghai Yien Chemical Technology Co., Ltd. (Shanghai, China). Baicalein (5,6,7-trihydroxyflavone, purity: 98%), LPS (*Escherichia coli* serotype 055: B5), paraformaldehyde (PFA), 4′,6-diamidino-2-phenylindole (DAPI), dichlorofluorescin diacetate (DCFDA) (purity ≥ 97%), bisbenzimide H33342 (purity ≥ 97%), and dimethyl sulfoxide (DMSO) were obtained from Sigma-Aldrich Co., Ltd. (Singapore). Antibodies used for Western blot were purchased from Cell Signaling Technology Inc. (Danvers, MA, USA). Primers were purchased from Integrated DNA Technologies Pte. Ltd. (Singapore).

### 4.2. Cell Culture

The macrophage cell line RAW 264.7 was obtained from the American Type Culture Collection (ATCC). Cells were cultured with Dulbecco’s Modified Eagle Medium (DMEM) supplemented with 10% fetal bovine serum (FBS) and 1% penicillin–streptomycin antibiotics and maintained in the 37 °C incubator with 5% CO_2_.

### 4.3. Cytotoxicity Test

RAW 264.7 cells were seeded in a 96-well plate at a density of 3 × 10^4^ cells/well and incubated overnight. Until wells filled with a monolayer, compounds were co-incubated with cells for 24 h. Afterwards, the cell viability was determined with a fresh mixture of cell counting kit 8 (Dojindo Molecular Technologies, Kumamoto, Japan) and DMEM (1:10, *v*/*v*) [9]. The absorbance at 450 nm was measured using the microplate reader (Tecan Infinite F200, Mannedorf, Switzerland). The cell viability was calculated as a percentage of the control group.

### 4.4. Quantitation of Nitric Oxide Concentration

RAW 264.7 cells were pretreated with 5,6-DHF at concentrations of 1, 5, 8.5, 10, 15, 25 and 30 μM for 1 h before the stimulation by LPS (100 ng/mL). After a 23 h incubation, the nitric oxide concentrations were quantified by Griess reagent (Promega, Singapore) according to the manufacturer’s instructions [9]. The absorbance was measured in a microplate reader at 540 nm using Tecan Infinite F200 (Tecan, Mannedorf, Switzerland). The normalized NO concentrations were calculated as a ratio to that of the DMSO group.

### 4.5. Western Blotting Analysis

RAW 264.7 cells were seeded in a 6-well plate at the density of 1 × 10^6^ cells/well and incubated overnight until cells formed a monolayer. After stimulation with LPS, cells were washed and lysed using the radio-immunoprecipitation assay buffer (Thermo Fisher Scientific, Singapore) at 4 °C for 15 min. Western blotting was conducted as reported before [9]. The blots were imaged by ChemiDoc Imaging System (Bio-Rad, Singapore) with Amersham ECL (Cytiva) or WesternBright™ Sirius (Advansta Inc., San Jose, CA, USA) and quantified by ImageJ 1.53a. All protein expressions were normalized to that of the DMSO group.

### 4.6. Real-Time Quantitative Polymerase Chain Reaction (qRT-PCR)

Total RNA of treated RAW 264.7 cells was extracted by Trizol reagent (Thermo Fisher Scientific) according to its protocols. The qRT-PCR process was conducted based on the reported method [17]. Primer sequences used were listed in Table 1. The RNA expression levels were analyzed by the $2^{-\Delta \Delta C_{T}}$ method with β-actin as the internal standard. All gene expressions were normalized to that of the DMSO group.

### 4.7. Determination of Cytoplasmic ROS and Mitochondrial ROS Levels

The cellular antioxidant activities were analyzed by reacting the cytoplasmic ROS with a redox-sensitive probe H_2_DCFDA. Macrophages monolayer in a 96-well plate was pretreated with 5,6-DHF at indicated concentrations for 1 h before the 23 h stimulation of LPS (100 ng/mL). Afterwards, H_2_DCFDA (10 μM) was incubated with cells at 37 °C for 15 min in the dark [41]. The endpoint fluorescence was measured at the wavelengths of 485 nm (excitation) and 535 nm (emission) using the BioTek Cytation 5 Cell Imaging Multimode Reader (Agilent, Santa Clara, CA, USA) after washing with PBS. 

Cells cultured on coverslips in a 24-well plate were subjected to the same treatment as the 96-well plate. The cell nuclei were stained with 10 μg/mL Hochest 33342 for 15 min. After washing to remove excessive dye, the cytoplasmic and mitochondrial ROS levels were determined by incubating cells with 10 μM H_2_DCFDA and 5 μM MitoSOX™ (Thermo Fisher Scientific) for 15 min in the incubator, respectively [42]. Glass coverslips were then transferred to a microscope slide and imaged under an Olympus CKX53 inverted microscope (EVIDENT, Tokyo, Japan) with an integrated 100 W mercury lamp (BH2-RFL-T2, Ushio, Tokyo, Japan) in the dark room. The images were captured and analyzed by EVIDENT cell Sens Entry 1.18.

### 4.8. Data Analysis

The multi-group data comparison was analyzed by Prism 10 using one-way analysis of variance (ANOVA). * *p* < 0.05 was considered statistically significant.

## 5. Conclusions

5,6-DHF blocked the activation cell surface stimuli receptor, hindered phosphorylation steps in MAPK, JAK-STAT, and NF-κB pathways, and downregulated pro-inflammatory mediators such as proteins and cytokines in the LPS-induced murine macrophage model. Its antioxidant function was revealed to act as a scavenger to eliminate cytoplasmic ROS and mitochondrial ROS and consequently alleviate oxidative stress. The overexpression of HO-1 and attenuation of inflammatory determinants by 5,6-DHF also contribute to the cellular redox balance. However, the specific cellular binding target of 5,6-DHF is still unclear and requires further investigation. Our findings demonstrated the potential application of 5,6-DHF as an anti-inflammatory and antioxidant agent and provide evidence for future investigation in animal models and humans.

## Figures and Tables

**Table 1:** Primer sequences for RT-qPCR.

Gene	Forward Primer	Reverse Primer
β-actin	5′-CCACAGCTGAGAGGGAAATC-3′	5′-AAGGAAGGCTGGAAAAGAGC-3′
IL-1β	5′-GGGCCTCAAAGGAAAGAATC-3′	5′-TACCAGTTGGGGAACTCTGC-3′
IL-6	5′-AGTTGC CTTCTTGGGACTGA-3′	5′-CAGAATGCCATTGCACAAC-3′
TNF-α	5′-AGCCCCCAGTCTGTATCCTT-3′	5′-CATTCGAGGCTCCAGTGAAT-3′

## Data Availability

All data presented this study are available from the corresponding author upon responsible request.
